# Unpacking the blood flow restriction device features literature: multi-chambered bladder design

**DOI:** 10.3389/fspor.2024.1457539

**Published:** 2024-10-10

**Authors:** Nicholas Rolnick

**Affiliations:** ^1^ Department of Exercise Science and Recreation, CUNY Lehman College, New York, NY, United States; ^2^ The Human Performance Mechanic, New York, NY, United States; ^3^ The BFR PROS, New York, NY, United States

**Keywords:** Delfi, multi-chambered, resistance training, limb occlusion pressure, BStrong, B3 Bands

## Introduction

Early training studies used single-chambered bladder tourniquets with arbitrary pressures (e.g., 100 mmHg) to study the impact of temporary blood flow restriction on muscle strength and hypertrophy (1, 2). Research since then has applied knowledge from medical tourniquet literature on factors that impact complete arterial occlusion including limb circumference, blood pressure, cuff shape and width, body positioning, and more (3, 4). Current practice recommendations indicate that personalizing the applied pressure (known as limb occlusion pressure, LOP) allows for a similar stimulus between participants in the same study and a better ability to compare different studies (5). Importantly, prescribing the pressure relative to the individual removes common methodological shortcomings and enhances safety (6). Literature indicates that the applied pressure used is an important methodological consideration given there appears to be a minimum amount of pressure (∼50% LOP) required to meaningfully accelerate muscular fatigue (7), a primary way BFR exerts its effect in practice. Therefore, it is crucial for BFR researchers and clinicians to point out BFR cuff features that may preclude LOP determination and the impact of such features on acute- and longitudinal outcomes of BFR.

In this manuscript, I discuss the challenges of a multi-chambered bladder design to the study of BFR exercise, focusing on the applied pressure prescriptions.

### The multi-chambered cuff: missteps in the literature

The growing interest in BFR exercise has led to new features that clinicians and researchers can use to modulate limb blood flow. Altering the design of the air bladder that applies circumferential pressure to the underlying limb is a potentially important device feature. Traditional BFR cuff bladders are single-chambered, meaning when air is pumped into the cuff it exerts a largely equal circumferential pressure around the limb, permitting a measurement of relativized pressure (8).

Recently, manufacturers (e.g., BStrong, B3 Bands) introduced a multi-chambered cuff designed to avoid arterial occlusion for participant safety (9). The design largely prevents arterial occlusion (and, consequently, the ability to determine a personalized pressure) due to the gaps between the sequential bladders, which do not compress the underlying limb (8). Reducing arterial inflow during resting conditions with multi-chambered cuffs occurs only at very high set pressures (e.g., the pressure set by the BFR user) (> 350 mmHg) (10). Thus, when researchers overlook this important cuff design, it can lead to conclusions not supported by single-chambered cuffs applied at the same pressure. As this body of evidence grows (11–19), it is important to highlight methodological oversights to reduce the likelihood of these occurring in future studies and in practice.

### Comparison of BFR cuff designs: impact on acute physiological and perceptual responses

Stray-Gunderson et al. (15) employed a short interval walking program (5 × 2 min at 3 mph) with bilateral Hokanson (single-chambered) or BStrong cuffs (multi-chambered) applied to the thighs. They determined that using a wide rigid Hokanson cuff (18 cm wide inflated to 160 mmHg) exacerbates the intra-exercise systolic blood pressure and double product (marker of myocardial demand) compared to the narrow elastic BStrong cuffs (5 cm wide) inflated to 300 mmHg. Additionally, the rating of perceived exertion and lactate release was significantly higher in the Hokanson condition whereas it was unchanged in the BStrong cuff condition. The authors concluded that the safety profile of BFR might be compromised with the Hokanson cuff and should be prescribed carefully.

However, it is perplexing that the authors did not attempt to relativize the pressure of the single-chambered cuff, especially considering the high usage of personalized pressures in practice (20) and the recommendations against arbitrary pressure use in research design (6). Although blood flow was not measured, the wide design of the Hokanson cuff and the inflation pressure would likely be near or exceed 100% LOP in most participants. For example, Hughes et al. (21) reported that a 13 cm-wide Hokanson [5 cm narrower than the cuff used in (15)] and reported that 163 mmHg was 100% LOP in their participant cohort (21). The use of such a wide cuff without determining a relativized pressure appears to have maximized the physiological stress of the exercise bout in the single-chambered cuff and elicit differences between cuffs. The BStrong cuffs behaved similarly to the low-intensity control exercise in most outcomes, except for a slightly greater increase in systolic blood pressure (15). This suggests the study may have been designed to favor the BStrong cuffs, as the widest cuffs recommended for practice (18 cm) (5) were applied with a higher-than-normal applied pressure in the Hokanson single-chambered cuff. Increased physiological demands are likely why low-intensity aerobic exercise with BFR is effective (22). As no longitudinal trials have investigated the impact of multi-chambered bladder cuffs on aerobic training outcomes, caution is warranted when extrapolating the effectiveness of aerobic exercise using cuffs with this feature, particularly given the limited acute physiological perturbations observed in the discussed study.

Bordessa et al. (11) compared muscle excitation and perceptual responses using the Delfi Personalized Tourniquet device (11.5 cm wide), applied at 80% LOP (∼152 mmHg), and the BStrong cuffs (5 cm wide), applied at ∼274 mmHg, during 30% 1RM leg extensions. These were compared against high-load control exercise at 80% 1RM. Across four fixed repetition sets (30-15-15-15), muscle excitation was similar between the BFR conditions, though both showed less excitation than high-load exercise. Additionally, the BStrong cuffs were perceived as less demanding than the Delfi Personalized Tourniquet, leading the authors to suggest that BStrong cuffs may be preferable in practice. However, it is well established that load, rather than applied pressure, is the primary driver of muscle excitation (23), so conditions exercising at a similar percentage of the one-rep max should produce comparable muscular excitation. Moreover, muscle excitation is not a reliable surrogate for hypertrophic potential during low-load exercise performed to high levels of voluntary effort as any low-intensity condition nearing failure is likely to produce similar long-term benefits, regardless of the BFR application (24). It is possible that participants using the BStrong cuffs were further from muscular failure compared to those using the Delfi cuffs, which may have led to lower perceived exertion despite the differing cuff pressures. This aligns with evidence that closer proximity to failure increases perceptual demands (25).

It is highly likely that the interface pressure (e.g., the pressure applied to the limb from the cuff) of the BStrong cuffs was significantly lower than that of the Delfi Personalized Tourniquet, despite differences in set pressures. This may also explain the higher perceptual demands, as pressure can modulate perceptual response (23). While both cuffs facilitate BFR exercise, the multi-chambered system modulates blood flow during resting conditions at pressures exceeding 350 mmHg, whereas the single-chambered cuff may require as little as 83 mmHg to achieve comparable effects (10).

It's important to note that the pressure applied with a multi-chambered cuff is not the same as the pressure applied to the underlying limb, as seen in traditional single-chambered cuffs (9, 26). This is a result of the multi-chambered design, where the set pressure does not directly correlate with the interface pressure (26). In fact, the multi-chambered system ensures that the device is unable to provide arterial occlusion even at pressures as high as 500 mmHg (9). Since no longitudinal studies exist using a multi-chambered cuff with fixed repetition schemes, it remains unclear whether the lower interface pressures would elicit meaningful hypertrophy compared to low-load exercise alone or BFR applied with a single-chambered cuff. This represents a critical area for future research, especially given the manufacturer's emphasis on safety, which may come at the cost of reduced effectiveness in BFR exercise due to the cuff's inability to sufficiently modulate blood flow compared to single-chambered cuffs.

### Misapplying algorithms meant for single-chambered cuffs while using multi-chambered cuffs

In 2014, Loenneke et al. introduced an algorithm based on thigh circumference using 5 cm wide elastic and nylon BFR cuffs to determine a ballpark estimate percentage of LOP without a doppler ultrasound or other automatic computer-based tourniquets (27). This algorithm is supposed to be applied when using cuffs of similar widths and bladder design to enhance the validity of application and ensure a sub occlusive BFR stimulus.

However, as BFR research has grown, researchers have begun to apply this algorithm inappropriately by not considering the impact of the bladder design on arterial occlusive capabilities (16, 19, 28). This is important from a methodological perspective because the interface pressure will be significantly less in the multi-chamber BFR cuff due to the bladder design despite a similar set pressure, potentially leading to conclusions or effects that differ when the same applied pressure is implemented with a single-chambered BFR cuff.

Wang et al. (16) and Zhang et al. (19) used Loenneke's limb circumference algorithm to determine restrictive pressure application, reporting average pressures between 180 and 260 mmHg using the BStrong cuffs (16, 19). Both studies demonstrated that adding BFR to high-load exercise and vibration training improved sports performance. However, the application of pressures in the range of 180-260 mmHg, particularly with multi-chambered systems like BStrong cuffs, may not produce the same level of blood flow restriction as single-chambered cuffs. The multi-chambered design likely results in lower interface pressures, potentially creating a compression-like effect rather than inducing true blood flow restriction (10, 29). It is important to note that blood flow modulation was not measured in these studies, limiting the ability to fully understand the exercise-induced effects of the applied pressure. While this compression may still enhance performance, it may not provide the same physiological stimulus as BFR using single-chambered cuffs, particularly regarding metabolic stress and occlusion-induced adaptations. If similar pressures were applied with a single-chambered cuff, it is likely that the response would differ, potentially reaching supra-occlusive levels. Future research should consider the impact of cuff design when determining applied pressure and prioritize the use of single-chambered cuffs when employing limb circumference algorithms to ensure a more consistent and effective BFR stimulus.

### Not considering pressure-dependent relationships when using multi-chambered cuffs

Research investigating pressure-dependent relationships in blood flow restriction has yielded mixed findings. Some studies suggest that arterial inflow is reduced in a non-linear fashion between 30 and 80% LOP in the brachial and superficial femoral arteries (30, 31), while others have found a more linear relationship in the posterior tibial artery (32). Despite these mixed results, it remains crucial to examine how pressure impacts the acute responses to BFR exercise. Single-chambered cuffs allow for a personalized, relativized pressure that can be tailored to everyone based on their LOP, ensuring a more consistent and effective BFR stimulus. The ability to adjust pressure to individual needs enhances the translation of research findings into practical applications. In contrast, multi-chambered cuffs such as BStrong cannot determine personalized pressure, as they do not significantly modulate blood flow from resting conditions unless very high pressures (>350 mmHg) are applied (10). This limitation reduces the ability to establish clear pressure-dependent relationships with this cuff design. Therefore, when studying pressure-dependent responses, single-chambered cuffs should be prioritized for their precision and the improved safety and efficacy they offer in BFR practice.

While single-chambered cuffs provide more precise pressure modulation and a clearer understanding of pressure-dependent responses, studies using multi-chambered cuffs like BStrong have begun to explore these relationships despite their inherent limitations. One such study by Jia et al. (13) investigated the pressure-dependent relationship of cerebral oxygenation levels following bilateral BFR squatting exercise using BStrong cuffs applied at pressures of 150, 200, 250, 300, and 350 mmHg. The authors found that cerebral oxygenation levels dropped sharply only at 350 mmHg. Based on this, they concluded that moderate applied pressures (150-250 mmHg) induced the most favorable acute responses in the cerebral cortex and should be used in BFR exercise. This recommendation was based on their observation that optimal cerebral activation and functional connectivity occurred at pressures between 150 and 250 mmHg, whereas 350 mmHg caused a significant decrease in oxygenated hemoglobin levels, indicating reduced cerebral blood flow. These results suggest that higher pressures, such as 350 mmHg, may lead to excessive restriction, reducing oxygenation and potentially diminishing the benefits of cerebral activation, which is crucial for neural adaptation during training.

However, it should be noted that in a separate study, 350 mmHg was the first pressure shown to modulate blood flow from resting conditions in the lower extremities with a multi-chambered cuff, achieving a similar effect to 40% LOP in a single-chambered cuff—the minimum recommended relativized pressure for BFR exercise (5, 10). While it might be tempting to equate 350 mmHg with 40% LOP due to their similar effects on blood flow reduction from resting conditions, without standardizing pressure relative to the individual, such comparisons remain speculative.

Additionally, given the existing knowledge on multi-chambered cuffs, the results of this study cannot be extrapolated to single-chambered cuffs. Applying the level of “moderate pressure” recommended by Jia et al. (13) in a single-chambered cuff would most certainly result in supra-occlusive pressure in most participants. Moreover, the multi-chambered bladder cuff is a poor choice to study pressure-dependent relationships in BFR exercise as it is designed to reduce the likelihood of arterial occlusion, not to function as a tourniquet (9). Thus, the results of Jia et al. (13) do not inform clinicians about how pressure impacts cerebral oxygenation responses when using more commonly applied single-chambered bladder BFR cuffs inflated to recommended 40%–80% LOP—especially as the only applied pressure that significantly impacted cerebral oxygenation was the minimum pressure that altered arterial blood flow from resting conditions (40% LOP) (10). Future research in this area should strongly consider how bladder design impacts restrictive capabilities, particularly if the desired response is a reduction in arterial blood flow.

## Conclusions and future directions

The current body of literature on multi-chambered BFR cuffs has several methodological oversights that can be addressed in future studies by taking the multi-chambered bladder design into account (Table 1). Given multi-chambered bladder cuffs are unlikely to fully occlude arterial blood flow, they are inherently unable to determine a personalized pressure, which significantly limits their ability to offer the same precision as single-chambered cuffs. As of mid-2024, no longitudinal studies have compared the chronic adaptive responses to fixed repetition schemes using multi-chambered cuffs, which would typically outperform low-intensity exercise when based upon the findings from single-chambered BFR cuffs. Therefore, while acute responses appear significantly diminished with multi-chambered BFR cuffs compared to single-chambered BFR cuffs, it remains unknown whether the long-term adaptations would differ. Understanding the impact of BFR cuff features, especially the limitations of multi-chambered cuffs in personalizing pressure, will be critical for advancing research and improving study designs. This will also better inform populations implementing multi-chambered BFR cuffs in their exercise routines.

**Table 1:** Differences between single- and multi-chambered BFR bladder designs.

	Single-chambered cuffs	Multi-chambered cuffs
Design	Single air bladder	Multiple sequential air bladders
Pressure application	Exerts equal circumferential pressure around the limb (traditional tourniquet)	Pockets of space between bladders reduce uniform pressure application
Personalized pressure (LOP) determination	Allows for personalized pressure determination and arterial occlusion	Often precludes personalized pressure determination due to design
Pressure levels	Effective at lower pressures given single-chambered design	Requires very high pressures (> 350 mmHg) to reduce arterial inflow from resting conditions
Research methodology	Commonly used in research with either arbitrary or personalized pressures	Methodological issues arise when using single-chamber algorithms on multi-chambered bladder cuffs
Physiological responses	Higher physiological stress and muscle excitation	Reduced physiological responses and muscle excitation
Perceptual responses	Higher rating of perceived exertion and lactate release	Lower perceptual demands and lactate release
Safety profile	Potentially higher risk if not personalizing the pressure to ensure sub occlusive stimulus	Designed for safety, reducing arterial occlusion risk
Impact on longitudinal outcomes	Effective in inducing hypertrophy and strength gains in a multitude of protocols	Unclear if effective in long-term adaptations given no research exists thus far and acute responses appear mixed as to whether it acts differently than low intensity exercise alone
Use in practice	Widely used with personalized pressures	Often used with arbitrary high pressures; consider that the high pressures are not necessarily being imparted on the underlying limb
